# Leukotriene B_4_ receptor locus gene characterisation and association studies in asthma

**DOI:** 10.1186/1471-2350-13-110

**Published:** 2012-11-20

**Authors:** Asif S Tulah, Bianca Beghé, Sheila J Barton, John W Holloway, Ian Sayers

**Affiliations:** 1 Division of Therapeutics and Molecular Medicine, University of Nottingham, Queen’s Medical Centre, Nottingham, United Kingdom; 2 Department of Oncology, Haematology and Respiratory Diseases, University of Modena & Reggio Emilia, Modena, Italy; 3 MRC Lifecourse Epidemiology Unit, Faculty of Medicine, University of Southampton, Southampton, United Kingdom; 4 Human Genetics and Medical Genomics, Human Development and Health, Faculty of Medicine, University of Southampton, Southampton, United Kingdom; 5 Present address: Institute of Cellular Medicine, Faculty of Medical Sciences, Newcastle University, Newcastle upon Tyne, United Kingdom

**Keywords:** Association, Asthma, Family based association test, Leukotriene, Leukotriene B_4_ receptor, RACE, Severity

## Abstract

**Background:**

Polymorphisms spanning genes involved in the production of leukotriene B_4_ (LTB_4_) e.g. *ALOX5AP* and *LTA4H* are associated with asthma susceptibility, suggesting a role for LTB_4_ in disease. The contribution of *LTB*_*4*_*receptor* polymorphism is currently unknown. The aim of this study was to characterise the genes for the two pivotal LTB_4_ receptors, *LTB4R1* and *LTB4R2* in lung tissue and determine if polymorphisms spanning these genes are associated with asthma and disease severity.

**Methods:**

Rapid amplification of cDNA ends (RACE) was used to characterise the *LTB4R1* and *LTB4R2* gene structure in lung. The *LTB4R1/2* locus on chromosome 14q11.2 was screened for polymorphic variation. Six *LTB4R* single nucleotide polymorphisms (SNPs) were genotyped in 370 Caucasian asthma families and 299 Adult Asthma Individuals (n=1877 total) and were evaluated for association with asthma and severity (BTS) outcome measures using Family Based Association Test, linear regression and chi square.

**Results:**

*LTB4R1* has complex mRNA arrangement including multiple 5′-untranslated exons, suggesting additional levels of regulation. Three potential promoter regions across the *LTB4R1/2* locus were identified with some airway cell specificity. 22 SNPs (MAF>0.01) were validated across the *LTB4R* locus in the Caucasian population. *LTB4R1* and *LTB4R2* SNPs were not associated with asthma susceptibility, FEV_1_ or severity.

**Conclusions:**

*LTB4R1* and *LTB4R2* shows splice variation in the 5′-untranslated region and multiple promoter regions. The functional significance of this is yet to be determined. Both receptor genes were shown to be polymorphic. *LTB4R* polymorphisms do not appear to be susceptibility markers for the development of asthma in Caucasian subjects.

## Background

Asthma is a multifactorial respiratory disease with genetic and environmental contributing factors. Leukotrienes are lipid mediators known to be involved in allergic conditions such as asthma and are generated by a series of enzymes and proteins which form the 5-lipoxygenase (5-LO) pathway [1]. There are two types of leukotriene; cysteinyl leukotrienes (CysLTs), which are potent bronchoconstrictors, and the dihydroxy leukotriene, leukotriene B_4_ (LTB_4_) a chemoattractant and activator of leukocytes. CysLTs have long been reported as important mediators contributing to inflammatory diseases such as asthma [2]. Recent data supports a role for LTB_4_ in asthma pathophysiology. LTB_4_ is elevated in the airways of asthma subjects and its concentration correlates with asthma severity [3,4].

Polymorphisms within genes encoding constituents of the 5-LO pathway provide excellent candidates for markers of asthma susceptibility. Polymorphisms in two 5-LO pathway genes; 5-lipoxygenase activating protein (*ALOX5AP)* and leukotriene A_4_ hydrolase (*LTA4H)* have shown an association with LTB_4_ overproduction from ionomycin-stimulated neutrophils and with myocardial infarction (MI) susceptibility [5,6]. 5-lipoxygenase activating protein (FLAP) is an adapter protein for the rate-limiting enzyme 5-lipoxygenase and is involved in the production of all leukotrienes; however LTA_4_H is specifically involved in LTB_4_ production*.* We and others have recently provided preliminary evidence that SNPs spanning *ALOX5AP* and *LTA4H* are asthma susceptibility markers and determinants of lung function [7,8]. Polymorphisms spanning *ALOX5*, *LTC4S,CYSLTR1* and *CYSLTR2* have also shown association with asthma-related traits, reviewed in [9].

Two G protein-coupled receptors (GPCRs) for LTB_4_ encoded by *LTB4R1* and *LTB4R2* have been described and are located together on chromosome 14q11.2 [10,11]. LTB_4_ via its receptors is important in the recruitment and activation of leukocytes to sites of inflammation [12] and these receptors have been proposed as potential therapeutic targets in asthma. Increasing our understanding of the expression, regulation and potential function of these receptors may provide important information for the design of therapeutic agents. Currently, relatively little is known about *LTB4R1* and *LTB4R2* gene structure, splice variation and polymorphic variation and the contribution of polymorphic variation to asthma and disease severity.

The aims of this study were to 1) investigate the gene structure of human *LTB4R1* and *LTB4R2* in cells and tissues relevant to asthma; 2) determine the extent and nature of polymorphic variation across the receptor locus and 3) determine if *LTB4R* polymorphisms were associated with asthma, lung function and disease severity in asthma families and adult asthma subjects. Our data suggest that *LTB4R1* and *LTB4R2* have complicated gene structure and are polymorphic and that polymorphisms spanning the *LTB4R* locus are not determinants of asthma susceptibility.

## Methods

### Cell culture and RNA/cDNA preparation

Human airway smooth muscle (HASM) cells were isolated and cultured as previously described [13]. Primary human bronchial epithelial cells (HBEC) were obtained from Lonza (Wokingham, UK) and cultured in bronchial epithelial growth medium (BEGM, Lonza, UK), using bronchial epithelial differentiation medium (BEDM, Lonza, UK) cells were differentiated at air-liquid interface [14,15]. The bronchial epithelial cell line BEAS-2B and a leukemic monocyte cell line, THP-1, were also cultured as described previously [14,16]. Commercial RNA for the lung, brain and placenta was obtained from Ambion (Huntingdon, UK) and peripheral blood mononuclear cells (PBMC), polymorphonuclear cells (PMN) was obtained from 3H Biomedical (Uppsala, Sweden). Cells were lysed and RNA extracted (from at least two different donors) using the RNeasy mini kit (Qiagen, Crawley, UK), as described by the manufacturer. cDNA was prepared using the Superscript first strand cDNA synthesis kit (Invitrogen, Paisley, UK) using random hexamers and 0.5-1.0μg total RNA per reaction, as directed by manufacturer.

### Rapid amplification of cDNA ends (RACE)

RACE was performed using the GeneRacer kit (Invitrogen) and Superscript II as directed [14]. RACE-ready lung cDNA was synthesised from 1μg total lung RNA obtained from Ambion (Huntingdon, UK) as described. Gene-specific primers were designed for 5′ and 3′ RACE in the coding region in an overlapping fashion. Plasmid DNA from RACE PCR clones was prepared using the DNA Miniprep kit (Qiagen) and sequenced with M13F and M13R vector primers using Big Dye v3.1 (Applied Biosystems) and an ABI 310 DNA sequencer. Sequence data was aligned to the human database using the Basic Local Alignment Search Tool (BLAST) 2 sequence alignment program.

### Polymorphism screening

The extended *LTB4R1* and *LTB4R2* genomic region identified by RACE (~11.5kb, NCBI build 37: +14:24776125–24787584) was amplified by multiple PCR reactions and screened for polymorphisms by direct sequencing using DNA extracted from whole blood of 35 individuals from the Nottingham Adult Asthma Cohort recruited on the basis of physician diagnosed asthma and no other respiratory illness with <10 pack-years smoking history. These subjects had severe asthma as defined by British Thoracic Society (BTS) step ≥ 3. Single nucleotide polymorphisms (SNPs) were identified by examining chromatograms and BLAST analysis of sequencing traces. Any potential SNP identified was validated by sequencing on the reverse strand of DNA. Ethical approval was obtained from the Nottingham University Hospitals local ethics committee.

### Subjects for association analyses

341 Caucasian families (n=1508) with at least two biological siblings with physician diagnosed asthma were recruited from the Southampton area. This cohort has been described in detail previously [17]. Baseline FEV_1_ (forced expiratory volume in one second) was measured as best of three values within 5% performed using Vitalograph dry-wedge bellows spirometer (Vitalograph Ltd, Buckingham, UK) and determined 14 days after respiratory tract infection or use of bronchodilator or anti-allergic medication. 46 Caucasian families (n=184) with at least two biological siblings with physician diagnosed asthma from the Nottingham area [17] were also recruited. Baseline lung function tests were performed, FEV_1_ defined as the best of three values. The Nottingham and Southampton cohorts were combined to generate a UK family cohort (n=370). A cohort comprising 299 unrelated adult European Caucasian individuals recruited from Nottingham and Padova was used [18]. Individuals were 16–60 years, had asthma for >1 year with no other respiratory illness and <10 pack-years smoking history. Baseline FEV_1_ was measured. All subjects were classified according to British Thoracic Society Step Guidelines (BTS steps, ranging from step 1 to step 5) based on physician prescribed medication [19]. Ethical approval was obtained from the Nottingham University Medical School, the Padova Local Ethics Committees and the Southampton and South West Hampshire and the Portsmouth and South East Hampshire Local Research Ethics Committees. Informed consent was provided by the adult (or parent/guardian for child subjects).

### SNP selection and genotyping

*LTB4R* SNPs were chosen for their ability to tag linkage disequilibrium (LD) blocks using Tagger software [20] or for inferred function. Sequencing data from the severe asthma subjects and available HapMap data (Build 36) were used to select the six SNPs for analysis. We acknowledge a limitation of the current study is the use of this available HapMap build that has now post SNP selection been superseded by 1000 genomes data. Six SNPs which captured the information for eight SNPs were selected. SNPs were also chosen due to potential functional significance. rs11158635 *(LTB4R2*, 5′UTR) tagged two SNPs (rs11158634 and rs2748543, both *LTB4R2*, 5′UTR) and rs3181384 (*LTB4R1*, 3′UTR) previously showed association with cardioembolic stroke in another study [21]. rs2224122 (*LTB4R2*, 5′UTR) was predicted as an intronic enhancer according to FASTSNP algorithm [22]. SNPs were genotyped by KBiosciences using KASPar (Hertfordshire, UK). Chi square was used to test for any deviation of the observed genotype frequency from the expected values under Hardy Weinberg Equilibrium. Allele frequencies between the UK and Italian subjects were not statistically different (data not shown).

### Association analyses

The family based association test (FBAT) software (version 1.5.1) [23] was used for association analyses in the family cohorts between *LTB4R* SNPs and phenotypic scores using the additive model. For analysis in the Adult Asthma Cohort, SPSS (version 15, SPSS Inc., Chicago, IL) was used to determine the contribution of each SNP to baseline percent predicted FEV_1_ using linear regression in the additive model. For dichotomised phenotypes, unadjusted contingency table analysis using the allelic model was completed using GraphPad Prism (version 5, San Diego, CA). Analyses in the adult asthma cohort were not corrected for any potential confounders to enable comparison of the association analysis between family-based and adult cohorts. Based on our study with six SNPs and three outcomes analysed, Bonferroni correction would suggest a p<0.003 when reporting results as statistically significant. There was between 0.814 (for a MAF of 0.096) and 0.990 (for a MAF of 0.494) power to detect an association with a significance level of p=0.05 and a relative risk of 1.5.

## Results

### Identification of *LTB4R1* and *LTB4R2* splice variants and promoter regions

**Figure 1:**
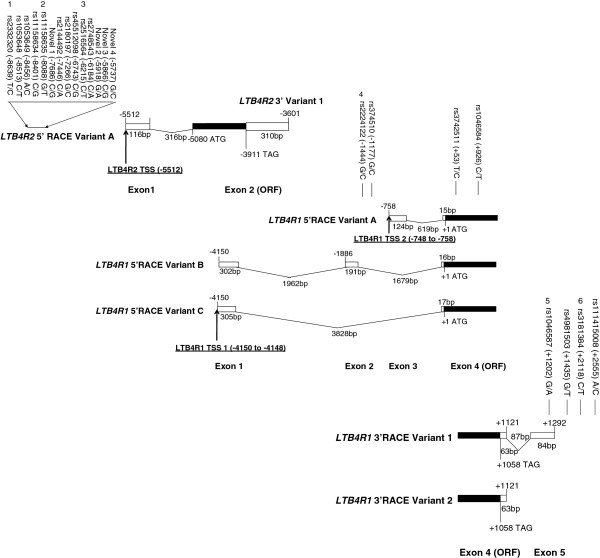
**Schematic representation of the human *LTB4R2 *and *LTB4R1 *genes on chromosome 14 showing overlapping gene structure and polymorphic variation.** Black boxes represent coding; *LTB4R2* (left), *LTB4R1* (right) and white boxes, non-coding exon sequence, space in-between represent intron. Data suggest *LTB4R1*/*LTB4R2* expression can be directed by at least three different promoter regions 5′ to the marked TSS (underlined). Validated polymorphisms in Caucasians are shown; positions relative to the *LTB4R1* ATG start codon (+1). SNPs numbered 1–6 correspond to genotyped SNPs in Table 3.

**Figure 2:**
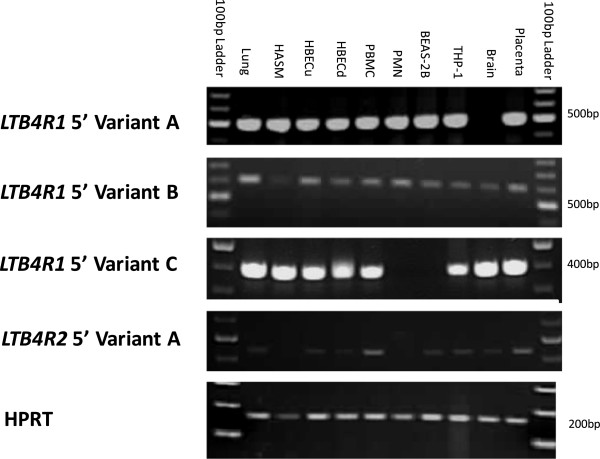
**Profiling *LTB4R1 *and *LTB4R2 *variants in lung and peripheral cells.** PCR was used to screen for the 5′RACE variants in various cells and tissues. A common reverse PCR primer was designed in the *LTB4R1* open reading frame sequence and different forward PCR primers were designed to assay for the four variants. RT- samples did not show any amplification signals (data not shown). HASM: human airway smooth muscle; HBECu: undifferentiated human bronchial epithelial cells; HBECd: differentiated human bronchial epithelial cells; PBMC: peripheral blood mononuclear cell; PMN: polymorphonuclear neutrophil; BEAS-2B: human bronchial epithelial cell line; THP-1: human acute monocytic leukemia cell line.

There was ubiquitous expression of *LTB4R1* and *LTB4R2* mRNA in the airway and periphery cells, including lung, HASM, HBEC, PBMC, PMN, BEAS-2B and THP-1 (data not shown). 5′ RACE data for *LTB4R1* and *LTB4R2* was generated for lung tissue (Figure 1). Three *LTB4R1* 5′ variants were identified by 5′ RACE in the lung which suggested a structure of four exons, with exon four (containing the protein coding region) being present in every variant identified. *LTB4R1* 5′ variant A was the most prevalent present in 50% of clones analysed (10 clones). A PCR-based assay validated this variant in lung, HASM, HBEC, PBMC, PMN, BEAS-2B and THP-1 (Figure 2). *LTB4R1* 5′ variant B was present in 35% of clones (7 clones). *LTB4R1* 5′ variant C was identified at lower frequency, 15% of clones (3 clones). *LTB4R1* 5′RACE data suggested the presence of two potential transcription start sites (TSS), at position −4150 to −4148 and one in the region −748 to −758 relative to the *LTB4R1* ATG. 3′ RACE data from the lung identified the presence of two variants; *LTB4R1* 3′ variant 1 present in 55% of clones (11 clones) and *LTB4R1* 3′ variant 2 was present in 45% of clones (9 clones). *LTB4R2* showed a more conserved structure based on RACE data. Only one 5′ structure was identified in all clones analysed (20 clones) which was validated in lung, HBEC, PBMC, BEAS-2B and THP-1 using PCR (Figure 2). The 3′ RACE data also showed a conserved structure, a 310bp untranslated region in all (n=20) clones analysed (Figure 1). We conducted an *in silico* analysis of the region 1.5kb upstream of the predicted three transcription start sites (data not shown). Multiple consensus sequences for Sp-1, AP-1 (activator protein 1), GATA, cAMP-response element binding protein, STAT (signal transducer and activator of transcription) and GR (glucocorticoid receptor) were identified.

### *LTB4R1* and *LTB4R2* sequencing

Direct sequencing in 35 individuals revealed 22 SNPs validated across the region (Table 1 and Figure 1). Four were novel SNPs and identified at low frequency (MAF <0.01) at position −7686, -5918, -5866, -5737 (in the *LTB4R2* 5′-untranslated region), all positions are relative to the *LTB4R1* ATG. 18 of these SNPs have now been reported (1000genomes and NCBI Build 37). Of note, the non-synonymous SNPs identified in Asian populations in *LTB4R1* (rs34645221, Ala79Ser and rs17849864, Leu346Phe) and *LTB4R2* (rs1950504, Asp196Gly) were not validated in the UK population analysed. Two synonymous coding region SNPs were identified, both in *LTB4R1*. rs3742511 (Ser18Ser) located in the extracellular N-terminus and rs1046584 (Gly309Gly) located in the cytosolic C-terminus.

**Table 1:** **SNPs identified from polymorphism screening in asthmatic subjects**.

**SNP**	**Alleles (major/minor)**	**Location**	**Gene location (amino acid)**	**Individuals sequenced (n)**	**Minor allele frequency**
rs2332320	T/C	−8639	*LTB4R2* 5′UTR	43	C=0.128
rs1053648	C/T	−8513	*LTB4R2* 5′UTR	43	T=0.035
rs1053649	A/C	−8456	*LTB4R2* 5′UTR	43	C=0.035
rs11158634	C/G	−8401	*LTB4R2* 5′UTR	43	G=0.244
rs11158635	G/T	−8088	*LTB4R2* 5′UTR	43	T=0.244
Novel 1	C/G	−7686	*LTB4R2* 5′UTR	35	G=0.014
rs2144492	C/A	−7446	*LTB4R2* 5′UTR	35	A=0.043
rs2180197	G/C	−7266	*LTB4R2* 5′UTR	35	C=0.043
rs45512098	C/G	−6743	*LTB4R2* 5′UTR	41	G=0.024
rs2516564	C/T	−6215	*LTB4R2* 5′UTR	41	T=0.22
rs2748543	C/A	−6184	*LTB4R2* 5′UTR	42	A= 0.27
Novel 2	G/A	−5918	*LTB4R2* 5′UTR	42	A=0.012
Novel 3	C/G	−5866	*LTB4R2* 5′UTR	42	G=0.012
Novel 4	G/C	−5737	*LTB4R2* 5′UTR	42	C=0.012
rs2224122	G/C	−1444	*LTB4R1* 5′UTR	43	C=0.163
rs374510	G/C	−1177	*LTB4R1* 5′UTR	44	C=0.011
rs3742511	T/C	+53	*LTB4R1* ORF (S/S)	39	C=0.026
rs1046584	C/T	+926	*LTB4R1* ORF (G/G)	39	T=0.28
rs1046587	G/A	+1202	*LTB4R1* 3′UTR	41	A=0.48
rs4981503	G/T	+1435	*LTB4R1* 3′UTR	41	T=0.11
rs3181384	C/T	+2118	*LTB4R1* 3′UTR	42	T=0.286
rs111415008	A/C	+2555	*LTB4R1* 3′UTR	41	C=0.012

The *LTB4R* locus was sequenced in 35 asthmatic subjects. 22 SNPs were validated, of which four were novel. Those SNPs with rs numbers correspond to those reported in the 1000genomes project and dbSNP (NCBI build 37).

### *LTB4R* polymorphisms are not associated with asthma, lung function or disease severity

**Figure 3:**
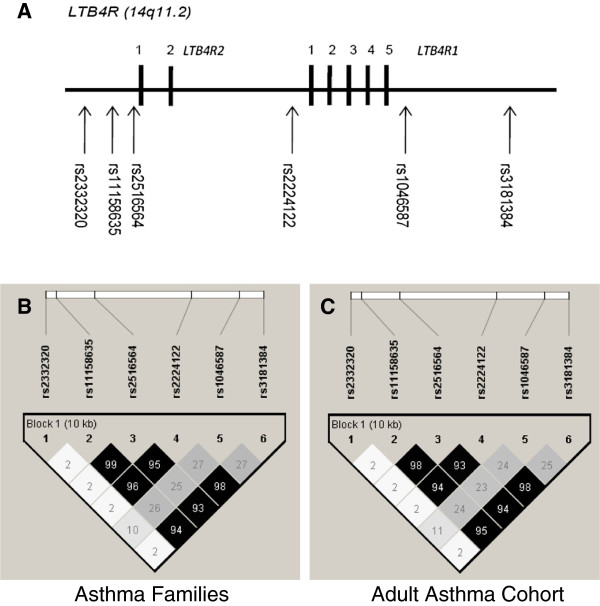
**Schematic diagram showing the location and linkage disequilibrium of *LTB4R *SNPs genotyped.** The LD plot shows the LD displayed as r^2^ in Haploview software [20]. Numerical values shown correspond to r^2^. **A**. represents the physical location of the SNPs genotyped. Black boxes represent exons and the spaces between introns determined by RACE. **B**. and **C**. represents the LD plot in the Asthma Families (n=370 families) and the Adult Asthma Cohort (n=299 individuals) respectively.

The clinical characteristics for all study cohorts are shown in Table 2. The family cohort contains children with asthma (mean age sibling 1 was 13.3±4.4 years and sibling 2 was 10.3±4.6 years) and the second asthma cohort is comprised of asthma adults (mean age 39.2±12.3 years, n=299). Genotyping data for the six SNPs did not show deviation from the Hardy-Weinberg Equilibrium (p>0.05). Minor allele frequencies (MAF) were similar across the different study cohorts. The haplotype structure of the *LTB4R* region generated using the genotyping data in the cohorts is shown in Figure 3. Analysis of LD across these SNPs indicated some redundancy in the genotyping with high LD between several *LTB4R* SNPs *e.g.* rs11158635, rs2516564 and rs2224122.

**Table 2:** Clinical characteristics of the study cohorts

	**Asthma Families**			**Adult Asthma Cohort**
	**Pedigrees**	**Sibling 1**	**Sibling 2**	
Age (years, mean±SD)	26.2±1.6	13.3±4.4	10.3±4.6	39.24±12.31
Gender (%, Female)	50.8	54.6	53.7	65.6
Asthma (%, Doctor diagnosed)	61.6	100	100	100
FEV_1_ (% Predicted) (mean±SD)	ND	95.4±15.6	96.2±14.9	92.37±20.57
Positive skin prick test (%)	62.5	73.9	65.1	64.3
Eczema (%, questionnaire)	41.1	53.5	55.1	31.8
Hay fever (%, questionnaire)	50.2	64.9	47.4	64.5
Log total serum IgE (mean)	2.04	2.33	2.33	2.03
Step on BTS guidelines (%):				
· Step 1	19.1	24.9	23.8	13.7
· Step 2	32.5	52.7	55.7	17.7
· Step 3	6.1	11.6	10.2	50.9
· Step ≥4	4.7	9.2	6.7	17.7
n	1578	370	361	299

Our data indicate no *LTB4R* SNPs tested were associated with asthma diagnosis, FEV_1_ or severity (BTS) in the families (Table 3). In the Adult Asthma Cohort we completed baseline percent predicted FEV_1_ analyses (Table 4), and again did not observe any significant associations. We retrospectively evaluated SNPs spanning *ALOX5AP* (8 SNPs) and *LTA4H* (6 SNPs) that encode for proteins involved in LTB_4_ production and had previously been associated with asthma susceptibility [7] with BTS defined severity (step 1–5). Interestingly, while no association survived correction for multiple testing, modest associations in the family cohort were observed with multiple *ALOX5AP* SNPs, e.g. SG13S41G (intron 4) (p=0.005, *z*=+2.778) and SG13S114A (intron 1) (p=0.017, *z*=+2.397). *LTA4H* SNP also showed modest association e.g., rs2540482C (5′UTR) (p=0.014, *z*=−2.468) (Table 5). Our previous study [7] analysed the effect of these same SNPs in determining asthma severity, but used an in house generated asthma severity score, which showed the same SG13S41G SNP with a p=0.021 and the same direction of effect as the present study. In the Adult Asthma Cohort dichotomised analysis of BTS 1 versus BTS ≥4 showed no significant association with any SNP tested (data not shown).

**Table 3:** ***LTB4R *SNP association analysis with asthma**, **FEV**_**1 **_**and BTS score 1 to 5 in 370 families**

**SNP no**.*	**SNP**	**Gene Location**	**Alleles**	**MAF**		**Asthma**			**FEV**_**1**_			**BTS (1–5)**	
					**Fam**	**Z-score**	**P-value**	**Fam**	**Z-score**	**P**-**value**	**Fam**	**Z-score**	**P-value**
	***LTB4R2***												
1	rs2332320	5′UTR	T/C	0.096	116	−0.173	0.863	121	−0.350	0.726	118	−1.000	0.317
2	rs11158635	5′UTR	G/T	0.211	202	−0.648	0.517	211	−0.813	0.416	204	+0.386	0.699
3	rs2516564	5′UTR	C/T	0.212	215	−1.109	0.268	221	−1.288	0.198	218	−0.060	0.952
	***LTB4R1***												
4	rs2224122	5′UTR	C/G	0.214	208	−0.907	0.365	217	−1.193	0.233	212	+0.103	0.918
5	rs1046587	3′UTR	G/A	0.494	282	−1.204	0.229	287	−1.391	0.164	287	−1.006	0.314
6	rs3181384	3′UTR	C/T	0.215	207	−0.895	0.371	215	−1.190	0.234	211	+0.157	0.875

The z score indicates the direction of the association (+ means the allele was overtransmitted = risk and – means undertransmitted = protection with respect to asthma affection; for continuous traits + indicates the allele confers higher trait values and – indicates the allele confers lower trait values). The z score is a measure of transmission equilibrium i.e. the deviation from the number of times an allele was transmitted to affected offspring and the number of times it should be transmitted under the null hypothesis of no association, no linkage. Number of families in analysis (≥10 families). *SNP location shown on Figure 1.

**Table 4:** **Baseline lung function** (**FEV**_**1**_) **and *LTB4R *SNPs in the adult asthma cohort**

**SNP**	**MAF**	**p-value (R**^**2**^**)**	**Group (n)**	**Value, % (Mean ± SEM)**
***LTB4R2***				
*rs2332320	0.10	0.703	0 (210)	92.568 ± 1.424
(5′UTR)		(0.001)	1 (50)	91.331 ± 2.918
rs11158635	0.19	0.421	0 (168)	93.592 ± 1.599
(5′UTR)		(0.007)	1 (79)	90.047 ± 2.331
			2 (11)	89.882 ± 6.247
rs2516564	0.19	0.422	0 (170)	93.529 ± 1.592
(5′UTR)		(0.007)	1 (77)	89.871 ± 2.365
			2 (10)	90.412 ± 6.564
***LTB4R1***				
rs2224122	0.20	0.599	0 (166)	93.469 ± 1.588
(5′UTR)		(0.004)	1 (81)	90.802 ± 2.274
			2 (12)	90.642 ± 5.907
rs1046587	0.49	0.761	0 (73)	90.804 ± 2.436
(3′UTR)		(0.002)	1 (118)	92.044 ± 1.916
			2 (67)	93.409 ± 2.543
rs3181384	0.20	0.694	0 (167)	93.157 ± 1.598
(3′UTR)		(0.003)	1 (82)	90.917 ± 2.281
			2 (12)	90.642 ± 5.962

Regression analysis was used to investigate the association between *LTB4R* SNPs and baseline percent predicted FEV_1_ in the Adult Asthma Cohort (n=299) using the additive model. 0, 1, 2 represent number of genotypes for major, heterozygotes and minor genotypes respectively. *Dominant model analysis was completed for rs2332320 due to low number of minor allele homozygotes in the additive model.

**Table 5:** ***ALOX5AP *and *LTA4H *SNP association analysis with BTS score 1 to 5 in 370 families**

**SNP**	**Gene Location**	**Alleles**	**MAF**	**Fam**	**Z-score**	**P-value**
***ALOX5AP***						
SG13S25	5′UTR	G/A	0.109	118	+0.977	0.329
SG13S114	Intron1	T/A	0.323	245	+2.397	0.017
rs3803277	Intron2	C/A	0.445	283	+1.818	0.069
SG13S89	Intron3	G/A	0.042	54	+1.886	0.059
rs4468448	Intron4	C/T	0.242	239	+1.183	0.237
SG13S32	Intron4	C/A	0.474	272	+1.617	0.106
SG13S41	Intron4	A/G	0.067	80	+2.778	0.005
SG13S35	3′UTR	G/A	0.078	97	+1.057	0.291
***LTA4H***						
rs1978331	Intron11	T/C	0.417	261	−1.865	0.062
rs17677715	Intron6	T/C	0.193	187	−1.974	0.048
rs2540482	5′UTR	T/C	0.222	209	−2.468	0.014
rs2660845	5′UTR	A/G	0.261	225	−0.856	0.392
rs2540475	5′UTR	C/T	0.215	212	−1.103	0.270

The z score indicates the direction of the association (for continuous traits + indicates the allele confers higher trait values and – indicates the allele confers lower trait values). The z score is a measure of transmission equilibrium i.e. the deviation from the number of times an allele was transmitted to affected offspring and the number of times it should be transmitted under the null hypothesis of no association, no linkage. Number of families in analysis (≥10 families).

## Discussion

Both LTB_4_R1 and LTB_4_R2 receptors are potentially important drug targets for conditions driven by inflammation involving LTB_4_. LTB_4_ production and activity is thought to be particularly important in severe asthma where a neutrophilic inflammation is more commonly observed [24]. The aims of this study were to characterise the *LTB4R1/2* locus at the molecular level to identify key regulatory regions (TSS, promoter regions), splice variation and polymorphic variation in lung tissue and to investigate the potential contribution of polymorphic variation to asthma susceptibility and severity. Our data show that *LTB4R1* and *LTB4R2* mRNA is ubiquitously expressed in multiple lung and peripheral cell types and that these genes are complex and have variation in 5′-untranslated regions and predicted promoter regions which may be functional in terms of cell-specific regulation. We also show that the *LTB4R1/2* locus is polymorphic (22 SNPs spanning ~11.5kb, MAF>0.01), with most variation in the untranslated regions. This study does not provide evidence supporting a role for *LTB4R* SNPs in susceptibility to develop asthma or severity phenotypes using asthma enriched families and adult asthma subjects. However, retrospective analyses of SNPs spanning *ALOX5AP* and *LTA4H* provided some evidence for association with BTS defined severity although this did not survive correction for multiple testing. This study represents the first characterisation of the *LTB4R* locus with respect to gene structure in the lung and the first evaluation of *LTB4R* SNPs for association with asthma susceptibility and severity.

LTB_4_ production is increased in asthma [3] with levels highest in severe asthmatics when compared to moderate asthmatics and control subjects [24]. The LTB_4_-LTB_4_R interaction is responsible for the influx of inflammatory cells into the lung. A significantly reduced recruitment of eosinophils and neutrophils into the airways has been demonstrated in mice deficient in *LTB4R* compared to wild type littermates [25]. Murine studies also show LTB_4_ is responsible for CD8+ T-cell mediated airway hyperresponsiveness through a mechanism involving mast cells [26]. These studies suggest LTB_4_ contributes to asthma pathogenesis through the recruitment and activation of neutrophils and eosinophils. Blocking the LTB_4_-LTB_4_R interaction with the inhibitor LY293111 led to a reduction in BAL neutrophils [27] and with the inhibitor U75302 reduced the migration and proliferation of airway smooth muscle cells which contributes to airway remodelling [28]. These data suggested a potential role in both the inflammatory and structural changes observed in the asthmatic airway.

Polymorphisms spanning *ALOX5AP* and *LTA4H* show association with LTB_4_ production from ionmycin stimulated neutrophils [5,6]. We have previously reported evidence that these same polymorphisms are associated with asthma susceptibility [7]. To date, little is known about the molecular structure of the two receptors for LTB_4_ (*LTB4R1* and *LTB4R2*) in lung and peripheral cells/tissues and regarding the effect of polymorphism contributing to asthma and severity phenotypes. Knowledge of the *LTB4R1* and *LTB4R2* isoforms and their expression pattern in effector cells will be useful when designing receptor antagonists and the effect of polymorphic variation across the receptors may show pharmacogenetic effects. We hypothesised that in addition to genes involved with LTB_4_ synthesis, alterations in genes encoding LTB_4_ target receptors may alter cellular responses to LTB_4_, such as inflammatory cell influx and contribute to asthma susceptibility and severity.

*LTB4R1* shows varied structure at the mRNA level with different 5′-untranslated structures and transcription start sites (TSS), whereas *LTB4R2* is more conserved. For *LTB4R1*, RACE identified a varied 5′-untranslated structure with three 5′-untranslated exons and one 3′-untranslated exon. This contrasted with previous findings suggesting three exons in the monocytic cell line THP-1 [29]. Our findings and those from Kato *et al.* supported the open reading frame (ORF) being contained in a single exon which showed concordance with other GPCRs showing intronless 5′ exons [29,30]. The homogeneity of these results between templates provides support for these variants being authentic. Previous studies have suggested *LTB4R1* contains one 5′-untranslated exon which is differentially spliced [10].

These different 5′-terminal exons give at least three different regions for transcriptional control across the *LTB4R* locus. Two different transcription start sites in *LTB4R1* at positions −4150 to −4148 and −748 to −758 and in *LTB4R2* at position −5512 (relative to *LTB4R1* ATG) were identified. -4150 has been reported in THP-1 cells and found to be active [29], supporting our finding of this TSS in the lung. The sequence for these transcription start sites were also identified in other lung and peripheral cells based on our PCR screen. Further characterisation of the promoter regions identified is needed to determine whether they are cell-specific. Our bioinformatic screen for transcription factor binding sites has shown multiple Sp-1 and AP-1 motifs in the promoter regions defined by TSS −5512 and −4150 to −4148, but not in the −748 to −758 region. Sp-1 is responsible for basal transcription and AP-1 is involved in inflammation, suggesting these promoter regions may be utilised under these conditions. The promoter region defined by −748 to −758 does not contain these motifs, but contains a GR (glucocorticoid receptor) site. GRs are transcription factors that are activated when bound to steroids. Activated GR can interact with other transcription factors, which can be positive (anti-inflammatory) or negative. The latter is observed in patients with steroid resistant asthma where transcription factors inactivate GR; a study has shown AP-1 may interfere with the binding of GR to DNA in steroid resistant patients [31]. Due to the close arrangement of *LTB4R1* and *LTB4R2* on chromosome 14q11.2 and the overlapping promoter region of *LTB4R1* being in the ORF of *LTB4R2*[11] raises the possibility that expression of these genes is regulated by the use of different promoters and may have cell-specific expression patterns. The complex 5′-untranslated region suggests transcriptional regulation may be important for tissue-specific regulation of the *LTB4R1* gene. This complexity can also lead to decreased efficiency of translation [32], which may be an important consideration when developing antagonists to target these receptors.

We screened the *LTB4R* locus to determine the level of polymorphic variation across the receptors in the Caucasian population. 22 SNPs were validated in our UK population. Previously suggested non-synonymous polymorphisms (which were identified in the Japanese population) in *LTB4R1* (rs34645221, Ala79Ser and rs17849864, Leu346Phe) and *LTB4R2* (rs1950504, Asp196Gly) [33] were not validated. 14 validated SNPs were at the 5′-end of the *LTB4R* locus, in the predicted *LTB4R2* promoter location. Of these 4 novel SNPs were identified. These had a low MAF (<0.05) and to date have not been reported in genetic databases or the 1000genomes browser. These could potentially affect transcriptional efficiency (of either *LTB4R2* or both *LTB4R1* and *LTB4R2* due to the close location of these genes). Our data also suggest strong linkage disequilibrium between the SNPs and a conserved nature of the locus which gives support for the two genes being formed by duplication during evolution [11].

Our studies did not observe any significant association with asthma, FEV_1_ or severity (BTS defined) in the asthma families or adult asthmatics for any *LTB4R* SNP analysed. These analyses suggest that these traits may not be genetically determined with respect to *LTB4R* polymorphism. Although there was no significant association, our data does show a constant direction of effect, suggesting this study may be underpowered to detect a subtle effect. Interestingly, there was modest evidence for a role of *ALOX5AP* and *LTA4H* SNPs associated with BTS defined asthma severity. These data therefore suggest genetically determined leukotriene production may be important in determining disease severity and not alterations in the downstream LTB_4_R receptor expression/activity. While no other study has assessed the role of *LTB4R* SNPs in asthma-related traits, research has involved SNPs spanning these genes in the cardiovascular field, where leukotrienes have also been shown to contribute to early atherosclerosis. rs1046587 and rs3181384 (both *LTB4R1*, 3′UTR) and three other SNPs spanning *LTB4R* were tested for association with carotid intima-media thickness in one study, but no association was observed after correction for multiple testing [34]. Also no association was observed with rs1046587 (*LTB4R1*, 3′UTR) and risk of ischemic stroke phenotypes in UK and German stroke cohorts [21]. This study did, however, identify significant or borderline association for the four other *LTB4R* polymorphisms tested: rs2748543 and rs3181384 (both in strong LD) with cardioembolic stroke in a UK cohort and rs1950505 and rs3742510 (also both in strong LD) with cardioembolic stroke in a German cohort [21]. Only rs2748543 was shown to be in LD with rs11158635 genotyped as part of this study.

Our study represents the first characterisation of the *LTB4R* locus with respect to gene structure in the lung and the first study to investigate association of *LTB4R* SNPs with asthma and importantly asthma severity where LTB_4_ has been suggested to have a more prominent role. We acknowledge the limitations of this study. One limitation was that RACE was conducted in the lung tissue which hampered our ability to detect cell-specific patterns of expression. Therefore it is likely that novel transcripts which may occur in other cells of the lung and periphery were not identified. To address some of these issues we profiled the variants identified by 5′RACE using PCR. Data suggest *LTB4R1* and *LTB4R2* show a ubiquitous expression profile in lung and peripheral cells and suggest that any antagonist targeting these receptors is unlikely to be cell-specific. We acknowledge that our sequencing cohort did not have the power to detect very rare SNPs (0.1%), however we did search the 1000genomes project resource to see if any of our identified rare variant SNPs (those with MAF<0.05) were validated by this project. Also there were modest numbers of families/individuals in our asthma cohorts used for association analyses. For this reason replication in larger additional cohorts is needed to validate our findings. Similarly, our asthma subjects had relatively preserved lung function which may have impeded our ability to detect association with FEV_1_ (% Predicted).

## Conclusions

In conclusion, this study has shown that *LTB4R1* and *LTB4R2* have complicated structure and are highly polymorphic. We also report the first evidence that SNPs spanning these genes are not associated with asthma, lung function or asthma severity.

## Abbreviations

5-LO: 5-lipoxygenase; BEDM: Bronchial epithelial differentiation medium; BEGM: Bronchial epithelial growth medium; BHR: Bronchial hyperresponsiveness; BLAST: Basic Local Alignment Search Tool; CYSLTs: Cysteinyl leukotriene; FBAT: Family based association test; FEV_1_: Forced expiratory volume in one second; FLAP: 5-lipoxygenase activating protein; GPCR: G protein-coupled receptor; HASM: Human airway smooth muscle; HBEC: Human bronchial epithelial cell; LD: Linkage disequilibrium; LTB_4_: Leukotriene B_4_; LTB4R1: Leukotriene B_4_ receptor 1; LTB4R2: Leukotriene B_4_ receptor 2; MI: Myocardial infarction; PBMC: Peripheral blood mononuclear cell; PMN: Polymorphonuclear neutrophil; RACE: Rapid amplification of cDNA ends; SNP: Single nucleotide polymorphism; TSS: Transcription start site; UTR: Untranslated region.

## Competing interests

The authors declare that they have no competing interests.

## Authors’ contributions

IS and AST designed the study and drafted the manuscript. AST completed the laboratory experiments and statistical analyses. BB, JWH and SJB were involved with cohort organisation. All authors contributed to the final version of the manuscript.

## Pre-publication history

The pre-publication history for this paper can be accessed here:

http://www.biomedcentral.com/1471-2350/13/110/prepub
